# Unraveling the structural and molecular properties of 34-residue levans with various branching degrees by replica exchange molecular dynamics simulations

**DOI:** 10.1371/journal.pone.0202578

**Published:** 2018-08-21

**Authors:** Surasak Chunsrivirot, Pongsakorn Kanjanatanin, Rath Pichyangkura

**Affiliations:** 1 Department of Biochemistry, Faculty of Science, Chulalongkorn University, Pathumwan, Bangkok, Thailand; 2 Structural and Computational Biology Research Group, Department of Biochemistry, Faculty of Science, Chulalongkorn University, Pathumwan, Bangkok, Thailand; Bioinformatics Institute, SINGAPORE

## Abstract

Levan has various potential applications in the pharmaceutical and food industries, such as cholesterol-lowering agents and prebiotics, due to its beneficial properties, which depend on its length and branching degree. A previous study also found that the branching degree of levan affected anti-tumor activities against SNU-1 and HepG2 tumor cell lines. Despite its promising potential, the properties of levans with different branching degrees are not well understood at the molecular level. In two models of the generalized Born implicit solvent (GB_HCT_ and GB_OBC1_), we employed replica-exchange molecular dynamics simulations to explore conformational spaces of 34-residue levans (L_34_) with branching degrees of zero (LFO_34B0_), one (LFO_34B1_), three (LFO_34B3_) and five (LFO_34B5_), as well as to elucidate their structural and molecular properties. To ensure a fair comparison of the effects of branching degree on these properties, we focused on analyzing the properties of the central 21-residue of the main chains of all systems. Our results show that all major representative conformations tend to form helix-like structures with kinks, where two-kink helix-like structures have the highest population. As branching degree increases, the population of helix-like structures with zero or one kink tends to increase slightly. As the number of kinks in the structures with the same branching degree increases, the average values of the lengths and angles among centers of masses of three consecutive turns of residue i, i+3, and i+6 tended to decrease. Due to its highest occurring frequencies, the O6 _(i)_—H3O _(i+1)_ hydrogen bond could be important for helix-like structure formation. Moreover, hydrogen bonds forming among the branching residue (br), branching position (bp) and other residues of L_34B1_, L_34B3_ and L_34B5_ were identified. The O1_(bp)_—H3O_(br)_, O1_(br)_—H3O_(br)_ and O5_(br)_—H1O_(br)_ hydrogen bonds were found in the first-, second- and third-highest occurrence frequencies, respectively. Our study provides novel and important insights into conformational spaces and the structural and molecular properties of 34-residue levans with various branching degrees, which tend to form helix-like structures with kinks.

## Introduction

**Fig 1:**
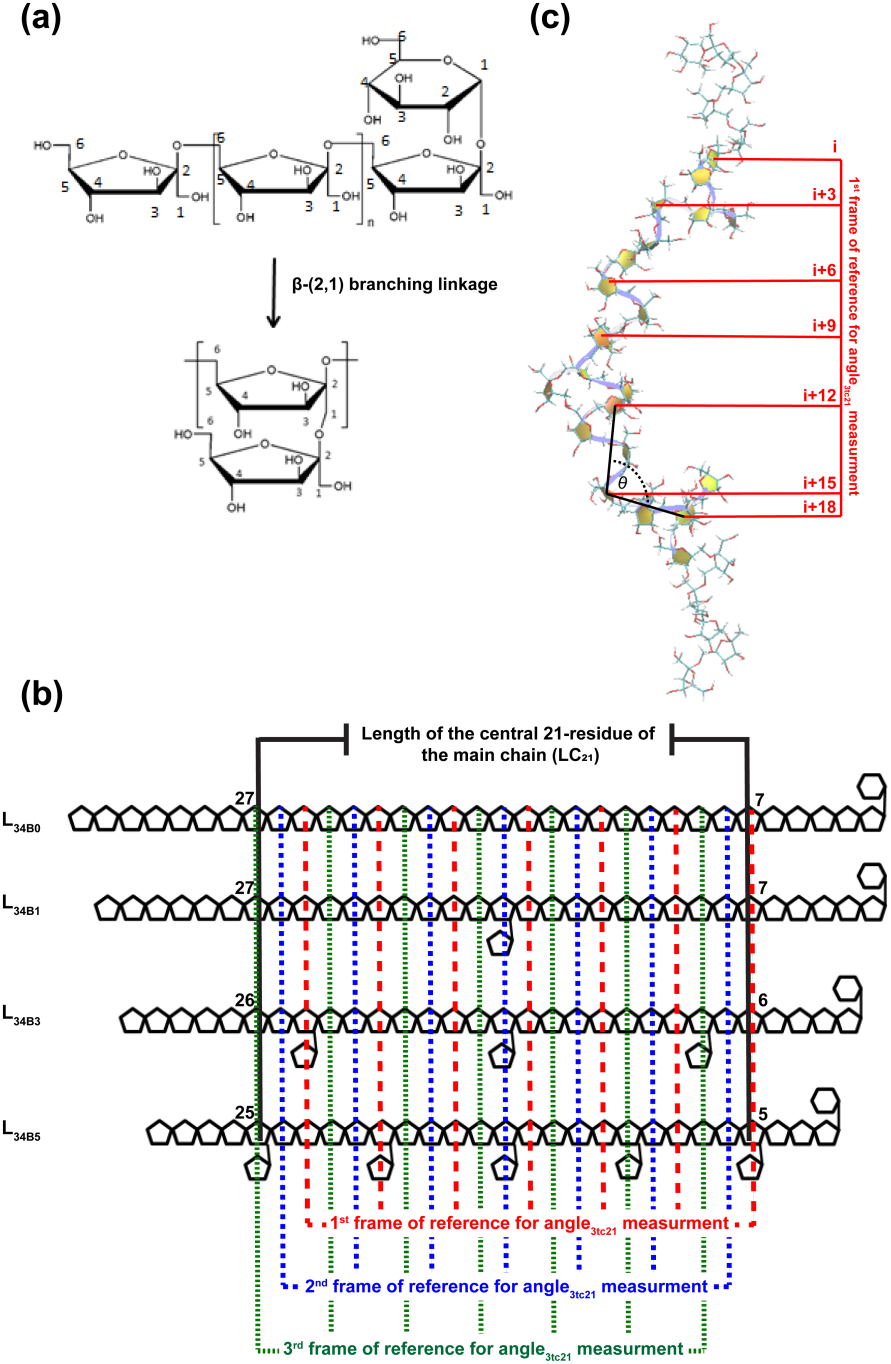
(a) Levan structure. (b) Simplified models of L_34B0_, L_34B1_, L_34B3_ and L_34B5_. LC_21_ and the first, second and third frames of reference for angle_3tc21_ measurement are also shown. (c) Example of angle_3tc21_ measurement for structure of L_34B3_, using the first frame of reference.

Levan is a polymer of fructose that is primarily connected through *β*-(2, 6) linkages in a main chain, with some *β*-(2, 1) linked branching points (Fig 1a). The production of levan can be performed using levansucrase from various organisms grown in sucrose-containing medium. As a member of the glycoside hydrolase family 68, levansucrase can catalyze a transfructosylation reaction to extend a levan chain and sucrose hydrolysis into glucose and fructose. Levansucrase can be found in various bacteria, including *Bacillus subtilis* [1], *Z*. *mobilis* [2], *Gluconacetobacter diazotrophicus* [3], *Pseudomonas syringae pv*. *Phaseolicola* [4], *Rahnella aquatilis* [5], and *Leuconostoc mesenteroides* [6].

Beneficial properties of levan include its unusually low intrinsic viscosity [7], high water solubility and susceptibility to acid hydrolysis [8], and these properties are very advantageous to various industries, especially the food and pharmaceutical industries. Examples of potential applications include cholesterol- and triacylglycerol-lowering agents [9], prebiotics [10], binders, controlled-release matrices [11], antiviral agents against avian influenza HPAI, H5N1 and adenovirus type 40 [12], as well as antitumor agents, whose activities depend on chain length and the degree of branching of levan [13,14]. A previous study by Yoon *et al*. found that the branching degree of levan affected the antitumor activity of levan on the SNU-1 and HepG2 tumor cell lines [14]. The antitumor activity of SNU-1 decreased as the branching degree of levan decreased from 12.3% to 4.2%. Additionally, the antitumor activity of HepG2 rapidly decreased as the branching degree changed from 12.3% to 9.3%. This antitumor activity then gradually increased as the branching degree decreased from 9.3% to 4.2%. Their results suggested the importance of the degree of branching on the structural properties of levan and its antitumor activities on the SNU-1 and HepG2 tumor cell lines [14]. Although levan has numerous potential applications, the structural and molecular properties of levan with various branching degrees are not well-understood at the molecular level.

To enhance the sampling accuracy and the probability of attaining the global minimum, the replica exchange molecular dynamics (REMD) technique simulates systems at various temperatures simultaneously and exchanges non-interacting systems among them [15,16]. Various studies have employed this method to explore the conformational spaces of oligosaccharides in solution, such as ε-cyclodextrin [17], high-mannose-type oligosaccharides [18], cellulose [19] and N-glycan core pentasaccharides [20]. Moreover, REMD was employed to investigate the conformational properties of a linear N-glycan [21], and a branched N-glycan found in the HIV envelop protein gp120 [21,22]. Recently, we used REMD to elucidate the structural and molecular properties of levan oligosaccharides (LFOs) with chain lengths of 5, 10 and 15 residues in two models of generalized Born implicit solvent (GB_HCT_ and GB_OBC1_) [23]. We found that LFOs tended to form helix-like structures as chain length increased from 5 to 15 residues [23]. However, to our knowledge, REMD has not been used to explore the conformational spaces of levan with various branching degrees in solution or to elucidate their properties.

In this study, we performed REMD on models of 34-residue levan (L_34_) with branching degrees of zero (L_34B0_), one (L_34B1_), three (L_34B3_) and five (L_34B5_) in two models of generalized Born implicit solvent (GB_HCT_ and GB_OBC1_). We aimed to explore their conformational spaces and elucidate their structural and molecular properties, as well as the relationship between these properties and the branching degree (Fig 1b). This knowledge may be beneficial for understanding how branching degree affects the structural and molecular properties of levan.

## Methods

### Structure preparation and minimization

The LEaP module in AMBER14 [24] was employed to build the structures of L_34B0_, L_34B1_, L_34B3_ and L_34B5_ and assign their atom types and force field parameters based on GLYCAM06j-1 [25] (Fig 1b). For minimization and simulation of each system, two implicit solvent models (GB_HCT_ and GB_OBC1_) were employed [26,27]. Minimization of all systems involved the 2,500 steepest-descent minimization cycles and 2,500 conjugate-gradient minimization cycles.

### Replica exchange molecular dynamics simulations

Initially, sixteen replicas per system were equilibrated for 500 ps to reach the desired temperature range from 284.0 to 584.5 K; these temperatures were distributed exponentially. Using the SANDER module in AMBER14, the REMD of each system was performed for 100 ns, and each replica was exchanged every 2 ps. The SHAKE algorithm was used to remove all bond-stretching freedoms associated with hydrogen, allowing a time step of 0.002 ps [28]. The random number generator was employed to reseed the initial velocity for all simulations [29]. A cut-off of 999 Å was employed to compute non-bonded interactions. To calculate the pairwise summation involved in the effective Born radii calculation, a maximum distance of 999 Å between atom pairs was used. To control the temperatures in all systems, Langevin dynamics with a collision frequency of 1 ps^-1^ were employed. The 100 ns trajectories of the replicas at 298 K were used for analysis of the structural and molecular properties of all systems.

To ensure a fair comparison of the effects of branching degree on the lengths of all systems, the lengths of the central 21-residue of the main chain (LC_21_) were measured (Fig 1b). Helix-liked structures with kinks were found with large populations in all systems, and each helical turn consisted of three fructosyl residues. To identify kinks and ensure a fair comparison of the effects of branching degree on the number of kinks in all systems, the angles among the centers of masses of three consecutive turns of residue i, i+3, and i+6 of the central 21-residue of the main chain (angle_3tc21_) were measured (Fig 1c). A kink was defined as an angle_3tc21_ less than 120°. The number of kinks was counted using three frames of reference, starting from the first, second or third residue of the central 21-residue of the main chain, respectively (Fig 1b and 1c). As counted by these three frames of reference, the median number of kinks was used to represent the number of kinks per structure. Free energy maps were plotted using the number of kinks and LC_21_ to characterize the structures of all systems. All structures were clustered based on the number of kinks. The structure most similar to the average structure of all members of each cluster was selected as a “centroid” to represent each cluster as a major representative conformer. This “centroid” is a structure with the lowest heavy-atom root-mean-square-deviation to the average structure.

To identify hydrogen bonds important for the formation of helix-like structures and hydrogen bonds involved with branching residues, the values of the occurrence frequencies of hydrogen bonds per structure and the occurrence frequencies of hydrogen bonds were calculated, respectively. Additionally, the occurrence frequencies of three dihedral angles between every two fructosyl residues of the main chains, ω (C4-C5-C6-O6), ψ (C5-C6-O6-C2’) and ϕ (C6-O6-C2’-O5’) were determined to measure conformational flexibilities of all systems.

## Results and discussion

### Reliability of REMD simulations

The acceptance ratios of the replica exchange were computed to verify that the temperatures were optimally distributed, and the number of replicas was sufficient. We found that the acceptance ratios of the simulations of L_34B5_ in the GB_HCT_ model were almost constant at approximately 29% (S1a Fig), implying a free random walk in the replica (temperature) space. Furthermore, our results show a free random walk both in the replica space (S1b Fig) and the temperature space (S1c Fig). Additionally, there is sufficient overlap between the canonical probability distribution of the total potential energy at each temperature and for those of neighbors (S1d Fig). Similar results were observed for the simulations of L_34B0_, L_34B1_ and L_34B3_ in the GB_HCT_ model. Their average acceptance ratios are almost constant at approximately 29%, 30% and 29% for L_34B0_, L_34B1_ and L_34B3_, respectively. Additionally, the results of REMD simulations of the systems simulated in the GB_OBC1_ model are also similar to those simulated in the GB_HCT_ model. Their average acceptance ratios are almost constant at approximately 29%. Our results demonstrate good reliability of the REMD simulations of all systems.

### Major representative conformers of L_34B0_, L_34B1_, L_34B3_ and L_34B5_

**Fig 2:**
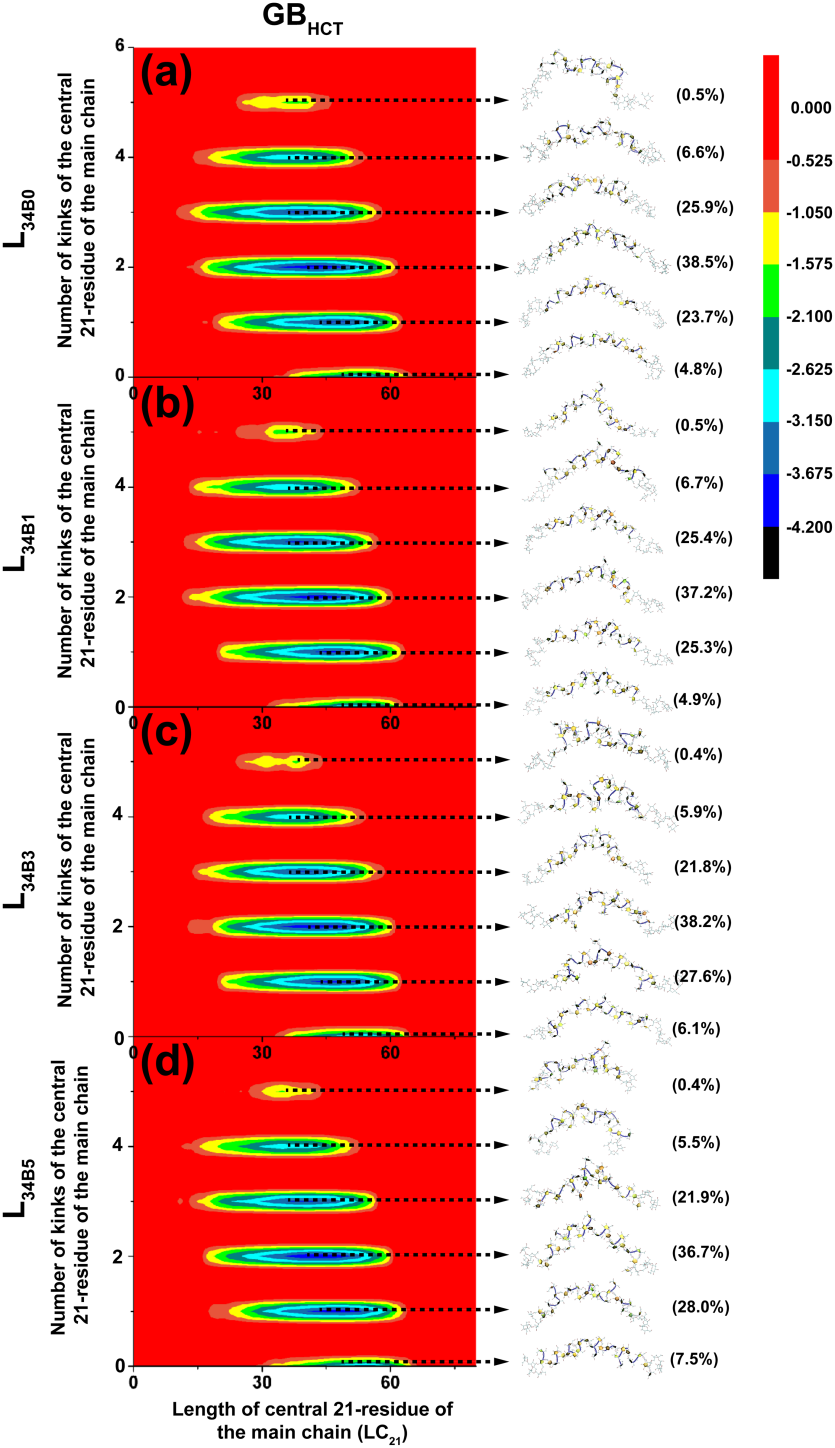
Relative free energy (kcal/mol) maps of L_34B0_ (a), L_34B1_ (b), L_34B3_ (c) and L_34B5_ (d) simulated in the GB_HCT_ model, as characterized by the number of kinks of the central 21 residue region of the main chain and LC_21_. Their major representative conformers and populations are also shown.

**Fig 3:**
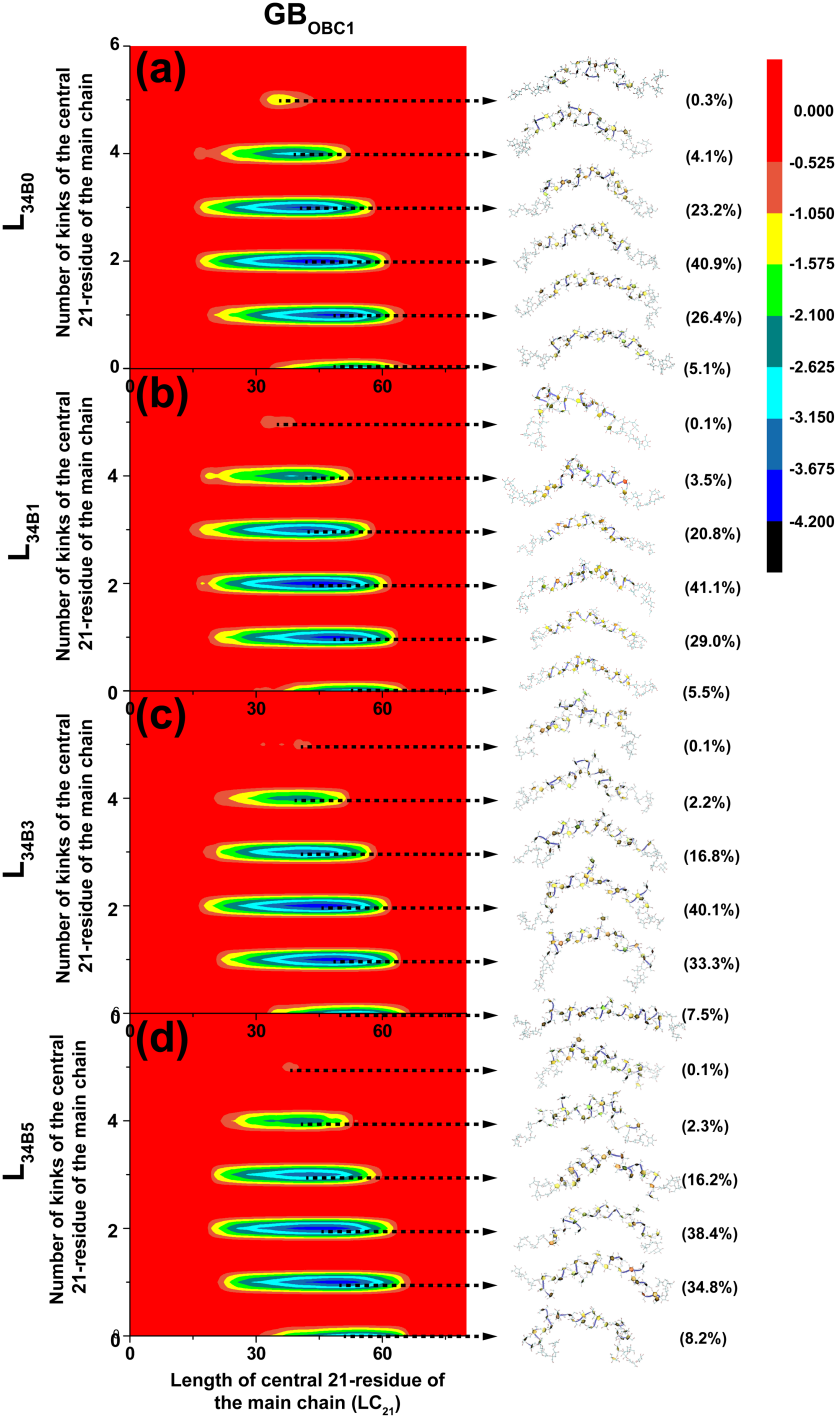
Relative free energy (kcal/mol) maps of L_34B0_ (a), L_34B1_ (b), L_34B3_ (c) and L_34B5_ (d) simulated in GB_OBC1_ model as characterized by number of kinks in the central 21 residue of the main chain and LC_21_. Their major representative conformers and populations are also shown.

To elucidate major representative conformers of L_34B0_, L_34B1_, L_34B3_ and L_34B5_, their free-energy maps were determined and shown with the major representative conformers and populations in Figs 2 and 3 for systems simulated in the GB_HCT_ and GB_OBC1_ model, respectively. These free-energy maps were characterized by the number of kinks in the central 21-residue portion of the main chain and LC_21_. These properties were measured at the central 21-residue of the main chains of all systems to ensure a fair comparison of the effects of branching degree on these properties (Fig 1b). All major representative conformers contain helical elements (helix-like structures), with the number of kinks ranging from zero to five. Helix-like structures adopted conformations of left-handed 3-fold helices, where each helical turn consisted of three fructosyl residues (Fig 1c). These results are consistent with previous findings that levan tends to form helix-like structures as its chain length increases from 5 to 15 residues [23]. In nature, kinks are also observed in DNA structures [30], long α-helical membrane proteins and soluble proteins (≥ 20 residues) [31]. Based on the number of kinks, all systems were clustered into six major representative conformers: helix-like structure, one-kink helix-like structure, two-kink helix-like structure, three-kink helix-like structure, four-kink helix-like structure and five-kink helix-like structure (Figs 2 and 3).

Table 1 shows that two-kink helix-like structures were found with the highest population of 38.5%, 37.2%, 38.2% and 36.7% for L_34B0_, L_34B1_, L_34B3_ and L_34B5_ simulated in the GB_HCT_ model, respectively. For structures with the same branching degree, the populations of major representative conformers tend to increase as the number of kinks increased from zero to two and subsequently decreased as the number of kinks increased from two to five. Moreover, our results show that as the branching degree increases from zero to five, the populations of helix-like structures and one-kink helix-like structures tend to increase slightly. Similar trends were also observed for those simulated in the GB_OBC1_ model (S1 Table). Additionally, we calculated the conformational distributions of the first and last 50 ns of the replica exchange molecular dynamics simulations. Examples of the conformational distributions of L_34B5_ in GB_HCT_ and GB_OBC1_ models are shown in S2 Table. These results show that the conformational distributions of the first 50 ns and last 50 ns simulations in each solvent model are reasonably similar, implying the convergence of the replica exchange molecular dynamics simulations.

**Table 1:** Populations of major representative conformers of L_34B0_, L_34B1_, L_34B3_ and L_34B5_ simulated in GB_HCT_ model. Ranges, highest frequency values, average values of LC_21_ and angle_3tc21_ are also shown.

Solvent model	Branch number	Number of kinks	Population (%)	LC_21_ (Å)			Angle_3tc21_ (°)		
				Range	Highest frequency (frequency)	Average (s.e.m.)	Range	Highest frequency (frequency)	Average (s.e.m.)
GB_HCT_	0 (L_34B0_)	0	4.8	30–66	54 (439)	52.2 (0.1)	40–180	155 (3891)	146.2 (0.1)
		1	23.7	18–68	48 (1337)	45.4 (0.1)	25–180	155 (14305)	135.7 (0.1)
		2	38.5	10–68	44 (2088)	41.1 (0.1)	20–180	140 (17749)	125.2 (0.1)
		3	25.9	10–62	40 (1402)	38.2 (0.1)	20–180	120 (13007)	115.1 (0.1)
		4	6.6	14–60	40 (397)	36.9 (0.1)	20–180	105 (4033)	106.0 (0.1)
		5	0.5	18–50	42 (44)	35.7 (0.3)	30–180	105 (387)	95.5 (0.4)
	1 (L_34B1_)	0	4.9	32–66	54 (383)	51.7 (0.1)	40–180	155 (3929)	145.1 (0.1)
		1	25.3	12–68	48 (1586)	45.6 (0.1)	30–180	150 (15184)	135.5 (0.1)
		2	37.2	12–62	44 (1981)	41.2 (0.1)	15–180	135 (17377)	125.2 (0.1)
		3	25.4	10–60	40 (1391)	37.9 (0.1)	15–180	120 (13048)	115.2 (0.1)
		4	6.7	14–56	36 (388)	35.5 (0.1)	25–180	115 (3665)	105.5 (0.1)
		5	0.5	16–48	35 (39)	34.3 (0.4)	25–180	105 (339)	97.2 (0.4)
	3 (L_34B3_)	0	6.1	28–68	54 (467)	52.0 (0.1)	35–180	155 (4898)	146.3 (0.1)
		1	27.6	14–64	48 (1639)	45.7 (0.1)	25–180	155 (16527)	135.7 (0.1)
		2	38.2	10–62	44 (2107)	42.0 (0.1)	20–180	145 (17771)	125.7 (0.1)
		3	21.8	14–62	40 (1289)	39.2 (0.1)	20–180	115 (11205)	115.7 (0.1)
		4	5.9	10–60	40 (389)	36.8 (0.1)	25–180	115 (3614)	106.2 (0.1)
		5	0.4	14–48	40 (38)	34.9 (0.4)	25–180	105 (303)	98.2 (0.4)
	5 (L_34B5_)	0	7.5	24–68	56 (645)	52.3 (0.1)	40–180	155 (6217)	146.0 (0.1)
		1	28.0	18–66	50 (1649)	46.1 (0.1)	20–180	155 (17028)	135.9 (0.1)
		2	36.7	16–66	44 (2012)	42.3 (0.1)	20–180	145 (17341)	125.6 (0.1)
		3	21.9	10–62	42 (1251)	39.2 (0.1)	25–180	120 (11410)	115.0 (0.1)
		4	5.5	12–58	42 (297)	35.9 (0.1)	25–180	120 (3204)	105.3 (0.1)
		5	0.4	20–48	36 (33)	35.2 (0.4)	30–180	105 (248)	96.5 (0.5)

### Distribution of LC_21_

To elucidate the effects of branching degree on the values of LC_21_, the distribution of LC_21_ was calculated and is shown in Table 1. As simulated in the GB_HCT_ model, the average values and highest frequency values of the LC_21_ of structures with the same branching degree tended to decrease as the number of kinks in the structures increases. These results indicate that the presence of kinks in a structure may cause the central 21-residue region of the main chain to be less extended, as its two ends could become closer each other. However, branching degree does not seem to significantly affect the values of LC_21_ because L_34B0_, L_34B1_, L_34B3_ and L_34B5_ appear to have similar ranges and trends of LC_21_ for all six major representative conformations, which have zero to five kinks. For all branching degrees, two-kink helix-like structures have the highest population with the highest frequency of LC_21_ value of 44 A°, and the average LC_21_ values are in the range of 41.1–42.3 A°. Similar trends were also observed in the structures simulated in the GB_OBC1_ model (S1 Table).

### Distribution of angle_3tc21_

To elucidate the effects of branching degree on the values of angle_3tc21_, the distribution of angle_3tc21_ was calculated and is shown in Table 1. As simulated in the GB_HCT_ model, the average and highest frequency values of angle_3tc21_ of the structures with the same branching degree tend to decrease as the number of kinks in the structures increases, due to the fact that kinks have low angle_3tc21_ (< 120°) values according to the definition in this study. However, branching degree does not seem to significantly affect the values of angle_3tc21_ because L_34B0_, L_34B1_, L_34B3_ and L_34B5_ appear to have similar ranges and trends for angle_3tc21_ values for all six major representative conformations, which have zero to five kinks. For all branching degrees, two-kink helix-like structures had the highest population with the highest frequency of angle_3tc21_ values in the range of 135–145°, with average angle_3tc21_ values in the range of 125.2–125.7°. Similar trends were also observed for the structures simulated in the GB_OBC1_ model (S1 Table).

### Hydrogen bonds that are important for formation of helix-like structures

**Fig 4:**
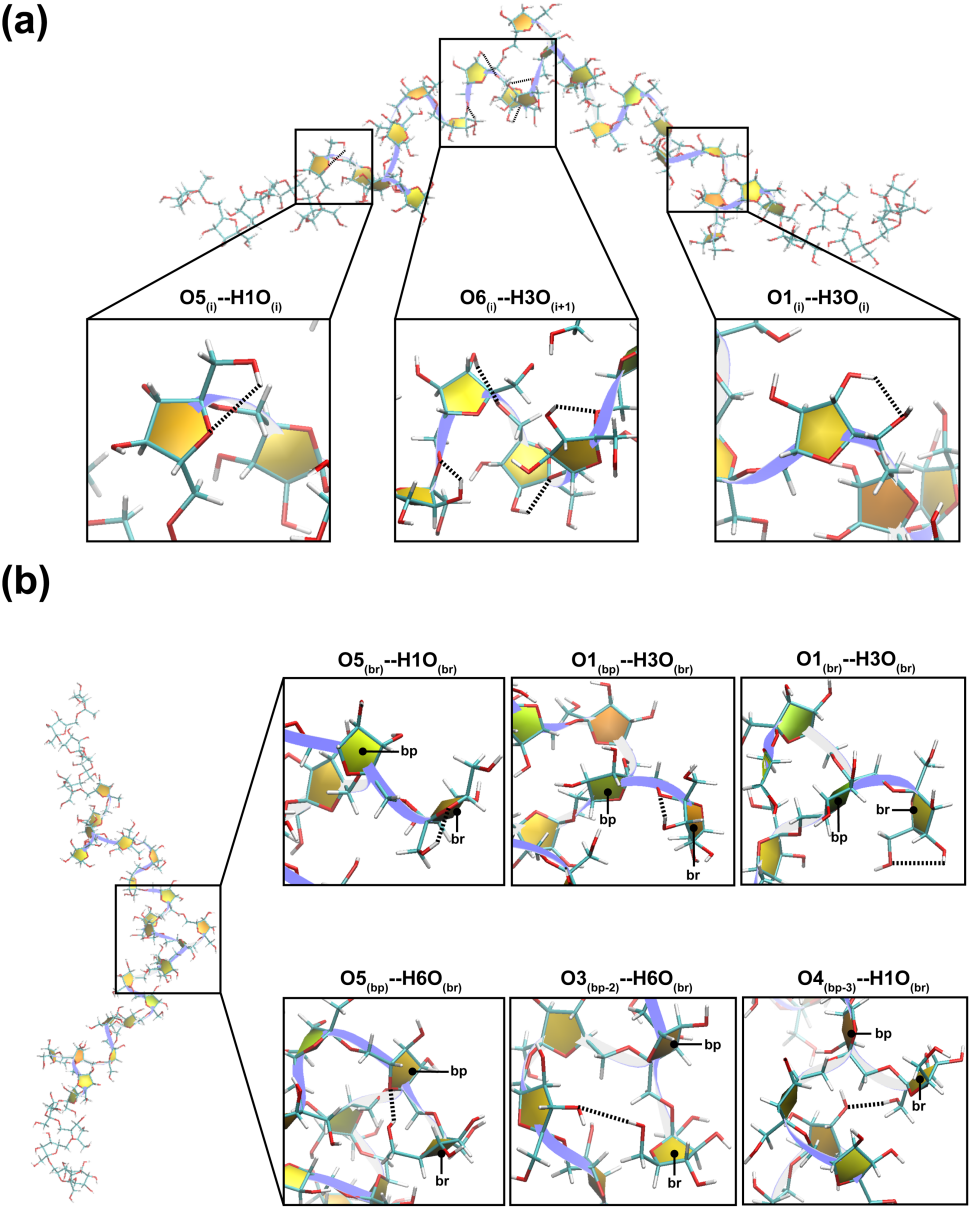
(a) Hydrogen bonds found in main chains. Middle; O6_(i)_—H3O_(i+1)_ hydrogen bond. Right; O1_(i)_—H3O_(i)_ hydrogen bond. Left; O5_(i)_—H1O_(i)_ hydrogen bond. (b) Hydrogen bonds involved in branching residues. The two-kink helix-like structure of L_34B3_ simulated in the GB_HCT_ model is shown as an example. Hydrogen bonds are represented as dashed lines. The levan chain and fructosyl units are represented as ribbons and filled yellow representations, respectively.

Since most structures contain helical elements, the occurrence frequencies of hydrogen bond per structure were calculated to identify hydrogen bonds important for the formation of helix-like structures. Hydrogen bonds with an occurrence frequency of hydrogen bonds per structure of at least 3% are shown in Table 2 and the S3 Table. The O6_(i)_—H3O_(i+1)_ hydrogen bond (between residue i and i+1) had the highest frequency for all systems simulated in the GB_HCT_ or GB_OBC1_ model. Its glycosidic oxygen acts as an important hydrogen bond acceptor that interacts with the hydroxyl groups of C3 atoms in the furanose ring and likely helps to stabilize a helix-like structure (Fig 4a). This hydrogen bond is likely to be important for the formation of a helix-like structure, as its occurrence frequency is significantly higher than that for other hydrogen bonds. For all systems simulated in the GB_HCT_ or GB_OBC1_ model, the O1_(i)_—H3O_(i)_ and O5_(i)_—H1O_(i)_ hydrogen bonds had the second and third highest frequencies, respectively; O1_(i)_—H3O_(i)_ and O5_(i)_—H1O_(i)_ hydrogen bonds are hydrogen bonds within the same residue (Table 2, S3 Table and Fig 4a). The trends for O6_(i)_—H3O_(i+1),_ O1_(i)_—H3O_(i)_ and O5_(i)_—H1O_(i)_ hydrogen bonds of these systems are similar to those found in the helix-like structures of LFO_10_ and LFO_15_ [23] probably because they all form helix-like structures, and these hydrogen bonds are likely to be important for helix formation. However, the occurrence frequencies of O6_(i)_—H3O_(i+1)_ hydrogen bond per structure of L_34B0_, L_34B1_, L_34B3_ and L_34B5_ are lower than those of the helix-like structures of LFO_10_ and LFO_15_. These results may be caused by the fact that L_34B0_, L_34B1_, L_34B3_ and L_34B5_ form curved helix-like structures and contain kinks in most of the structures; therefore, it is more difficult for O6_(i)_—H3O_(i+1)_ hydrogen bonds to form. On the other hand, LFO_10_ and LFO_15_ form helix-like structures that are less curved than L_34B0_, L_34B1_, L_34B3_ and L_34B5_ because they have less number of residues than L_34B0_, L_34B1_, L_34B3_ and L_34B5_ and they are less flexible and too short to form kinks. As a result, O6_(i)_—H3O_(i+1)_ hydrogen bonds are more likely to form in helix-like structures of LFO_10_ and LFO_15_.

**Table 2:** Occurrence frequency of hydrogen bonds per structure of L_34B0_, L_34B1_, L_34B3_ and L_34B5_ simulated in GB_HCT_ model.

Solvent model	Branch number	Number of kinks	Occurrence frequency of hydrogen bonds per structure (%)*		
			Between residue i, i		Between residue i, (i+1)
			O5_(i)_—H1O_(i)_	O1_(i)_—H3O_(i)_	O6_(i)_—H3O_(i+1)_
GB_HCT_	0 (L_34B0_)	0	8.3	9.9	43.0
		1	8.8	9.9	43.4
		2	8.9	9.6	43.0
		3	9.4	9.6	42.2
		4	9.4	9.0	41.9
		5	10.2	8.0	38.1
	1 (L_34B1_)	0	8.7	11.1	42.3
		1	8.0	10.3	43.1
		2	8.7	10.2	42.9
		3	8.6	10.2	42.8
		4	8.0	9.9	43.5
		5	8.8	9.8	38.1
	3 (L_34B3_)	0	6.7	11.6	46.2
		1	7.4	11.3	43.9
		2	7.6	11.5	43.2
		3	8.1	11.6	42.1
		4	7.8	12.2	41.3
		5	7.2	12.4	46.0
	5 (L_34B5_)	0	6.3	9.4	45.4
		1	6.7	11.1	44.2
		2	6.8	11.9	43.1
		3	6.7	11.5	42.5
		4	6.7	13.0	41.1
		5	7.4	14.8	40.7

*Only hydrogen bonds with occurrence frequency per structure of at least 3% are shown.

As branching degree increases, the occurrence frequency of hydrogen bonds per structure for the O5_(i)_—H1O_(i)_ hydrogen bond tends to decrease, while that of O1_(i)_—H3O_(i)_ hydrogen bonds tend to increase for systems simulated in the GB_HCT_ or GB_OBC1_ model (Table 2 and S3 Table). These trends are likely caused by the fact that H1O_(i)_ is removed at each branching position to build an O1 glycosidic linkage with a branching residue. Therefore, less H1O_(i)_ are available for hydrogen bond formation with O5_(i)_, but more O1_(i)_ are available for hydrogen bond formation with H3O_(i)_.

### Hydrogen bonds involved with branching residues

To identify hydrogen bonds involved with branching residues, the occurrence frequencies of hydrogen bonds forming among branching residues (br), branching position (bp) and other residues of L_34B1_, L_34B3_ and L_34B5_ were measured. The occurrence frequencies of these hydrogen bonds are drastically lower than those forming in the main chains (Table 3 and S4 Table) because the number of branching residues is significantly less than the number of residues in the main chains. For all systems simulated in the GB_HCT_ or GB_OBC1_ model, the O1_(bp)_—H3O_(br)_ hydrogen bond between the glycosidic oxygen of the branching position and the hydroxyl group of the C3 atom of the furanose ring of the branching residue were found at the highest frequency (Table 3, S4 Table and Fig 4b). Moreover, the O1_(br)_—H3O_(br)_ and O5_(br)_—H1O_(br)_ hydrogen bonds were found with the second and third highest frequencies, respectively (Table 3, S4 Table and Fig 4b). These O1_(br)_—H3O_(br)_ and O5_(br)_—H1O_(br)_ hydrogen bonds are hydrogen bonds within the same residue, which are similar to those found in the main chain (O1_(i)_—H3O_(i)_ and O5_(i)_—H1O_(i)_ hydrogen bonds). O5_(bp)_—H6O_(br),_ O3_(bp-2)_—H6O_(br)_ and O4_(bp-3)_—H1O_(br)_ hydrogen bonds were also observed. The formation of O1_(bp)_—H3O_(br)_ and O5_(bp)_—H6O_(br)_ hydrogen bonds are probably caused by the fact that H1O_(bp)_ were removed at a branching position to build an O1 glycosidic linkage with a branching residue. Therefore, the O5_(bp)_—H1O_(bp)_ hydrogen bond could not be formed in the main chain. As a result, O1_(bp)_ was available to form a hydrogen bond with H3O_(br)_, while O5_(bp)_ was available to form a hydrogen bond with H6O_(br)_.

**Table 3:** Occurrence frequency of hydrogen bond involved with branching residues of L_34B1_, L_34B3_ and L_34B5_ simulated in the GB_HCT_ model.

solvent model	Branch number	Branching position	Occurrence frequency of hydrogen bond (%)*					
			Between the same residue		With other residues			
			O1_(br)_—H3O_(br)_	O5_(br)_—H1O_(br)_	O1_(bp)_—H3O_(br)_	O5_(bp)_—H6O_(br)_	O3_(bp-2)_—H6O_(br)_	O4_(bp-3)_—H1O_(br)_
GB_HCT_	1 (L_34B1_)	17	0.33	0.24	0.89	0.18	0.13	0.11
	3 (L_34B3_)	8	0.31	0.26	0.88	0.18	0.12	0.10
		16	0.30	0.26	0.88	0.21	0.13	0.10
		24	0.32	0.24	0.90	0.20	0.12	0.12
	5 (L_34B5_)	5	0.26	0.25	0.81	0.19	0.16	0.11
		10	0.32	0.25	0.82	0.20	0.13	0.11
		15	0.30	0.25	0.87	0.18	0.14	0.10
		20	0.32	0.22	0.81	0.18	0.15	0.13
		25	0.30	0.26	0.86	0.19	0.10	0.09

*Only hydrogen bonds with occurrence frequency of at least 0.05% are shown.

### Conformational flexibilities

**Fig 5:**
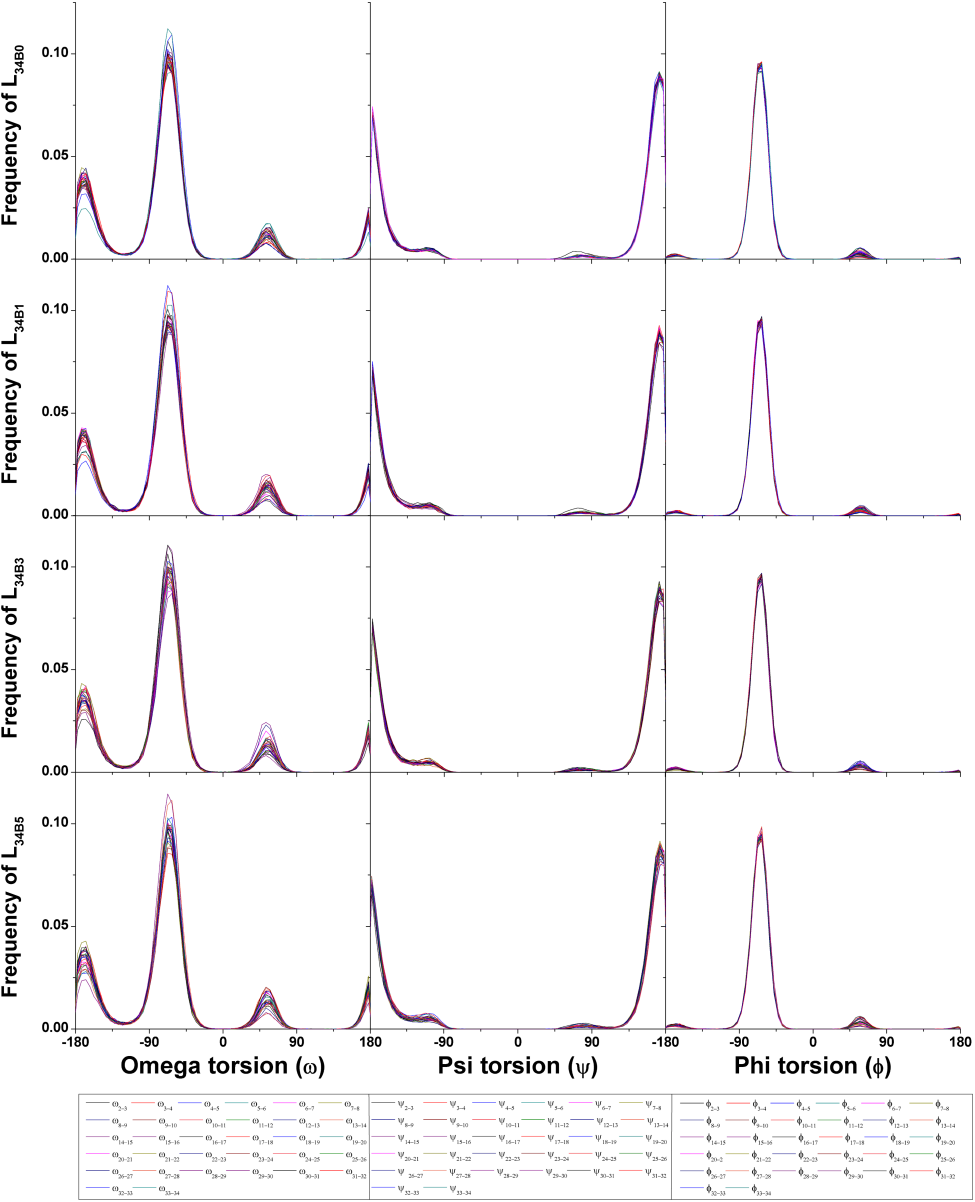
The occurrence frequencies of ω, ψ and ϕ between every two fructosyl residues of LFO_34B0_, LFO_34B1_, LFO_34B3_ and LFO_34B5_ simulated in the GB_HCT_ model. Each dihedral angle is shown in different color.

To measure conformational flexibilities of all systems, the occurrence frequencies of ω, ψ and ϕ of the main chains were computed (Fig 5 and S2 Fig). The value of ω with the highest frequency is around -65° to -60° (major peak). Moreover, there are two additional peaks centering around -170° to -165° and 55° to 60°, respectively. The major peak of ψ centers around 175° to 180° with two shoulders. The first shoulder starts from ψ value around -65°, and the second shoulder starts from ψ value around 40° or 45° for the systems simulated in the GB_HCT_ or GB_OBC1_ model, respectively. The major peak of ϕ centers around -65° to -60°. There are also two very small peaks centering around -170° to -165° and 60° to 65°. These results show that ω is probably more flexible than ψ and ϕ because it has more possibilities in rotating and changing levan conformations. The trends of the occurrence frequencies of ω, ψ and ϕ of the main chains of these systems are similar to those of levan oligosaccharides with the chain lengths of 5, 10 and 15 residues [23].

## Conclusions

To explore the conformational spaces of 34-residue levans with various branching degrees and elucidate their structural and molecular properties and the effects of branching degree on these properties, we performed REMD on 34-residue levans with branching degrees of zero, one, three and five. To ensure a fair comparison of the effects of branching degree on the structural and molecular properties, we focused on analyzing the properties of the central 21-residue region of the main chains. Our results show that all major representative conformations of all branching degrees tend to form helix-like structures with kinks, and two-kink helix-like structures had the highest population. Moreover, our findings reveal that as the branching degree increases from zero to five, the populations of helix-like structures and one-kink helix-like structures tend to increase slightly. Furthermore, the average values and highest-frequency values of LC_21_ and angle_3tc21_ for structures with the same branching degree tend to decrease as the number of kinks in the structures increases. For all systems, the O6_(i)_—H3O_(i+1)_ hydrogen bond has the highest occurrence frequency per structure and is likely to be important for the formation of a helix-like structure, as its frequency is substantially higher than other hydrogen bonds. Hydrogen bonds involved with branching residues were found with substantially lower frequencies than those important for helix-like structure formation. Examples of these hydrogen bonds are the O1_(bp)_—H3O_(br)_, O1_(br)_—H3O_(br)_ and O5_(br)_—H1O_(br)_ hydrogen bonds that were found with the first, second and third highest frequencies, respectively. It is worth mentioning that GB implicit solvent models may not be able to model highly specific solute-water interactions as accurate as explicit solvent model and may affect conformational sampling. Our study employed GB implicit solvent models because it is currently computationally prohibitive to perform REMD on 34-residue levans in explicit solvent model. Furthermore, GB implicit solvent models were successfully employed to explore conformational spaces of oligosaccharides such as *ε*-cyclodextrin [17] and levan oligosaccharides [23]. This work provides important and novel insights into the structural and molecular properties of levan with various branching degrees at the molecular level, as well as the effects of branching degree on these properties.

## Supporting information

S1 Fig Reliability of REMD simulations. (a) Acceptance ratio of replica exchange of adjacent pairs in simulations of L_34B5_ in the GB_HCT_ model. (b) Replica exchange at 298 K. (c) Time series of temperature exchange of three arbitrarily chosen replicas 1 (black), 9 (red) and 15 (blue). (d) Canonical probability of total potential energy of 16 temperatures for simulations in GB_HCT_ model.
(TIF)

S2 Fig The occurrence frequencies of ω, ψ and ϕ between every two fructosyl residues of LFO_34B0_, LFO_34B1_, LFO_34B3_ and LFO_34B5_ simulated in the GB_OBC1_ model. Each dihedral angle is shown in different color.
(TIF)

S1 Table Populations of major representative conformers of L_34B0_, L_34B1_, L_34B3_ and L_34B5_ simulated in GB_OBC1_ model. Ranges, highest frequencies, averages of LC_21_ and angle_3tc21_ are also shown.
(DOC)

S2 Table Conformational distributions of the first and last 50 ns of the replica exchange molecular dynamics simulations of L_34B5_ in GB_HCT_ and GB_OBC1_ models. (DOC)

S3 Table Occurrence frequency of hydrogen bond per structure of L_34B0_, L_34B1_, L_34B3_ and L_34B5_ simulated in the GB_OBC1_ model. (DOC)

S4 Table Occurrence frequency of hydrogen bonds involved with branching residues of L_34B1_, L_34B3_ and L_34B5_ simulated in the GB_OBC1_ model. (DOC)
