# Supplementary figures and images for: Unraveling the structural and molecular properties of 34-residue levans with various branching degrees by replica exchange molecular dynamics simulations
Source: PLoS One. 2018 Aug 21;13(8):e0202578. doi: 10.1371/journal.pone.0202578 (PMC6103501; doi:10.1371/journal.pone.0202578)

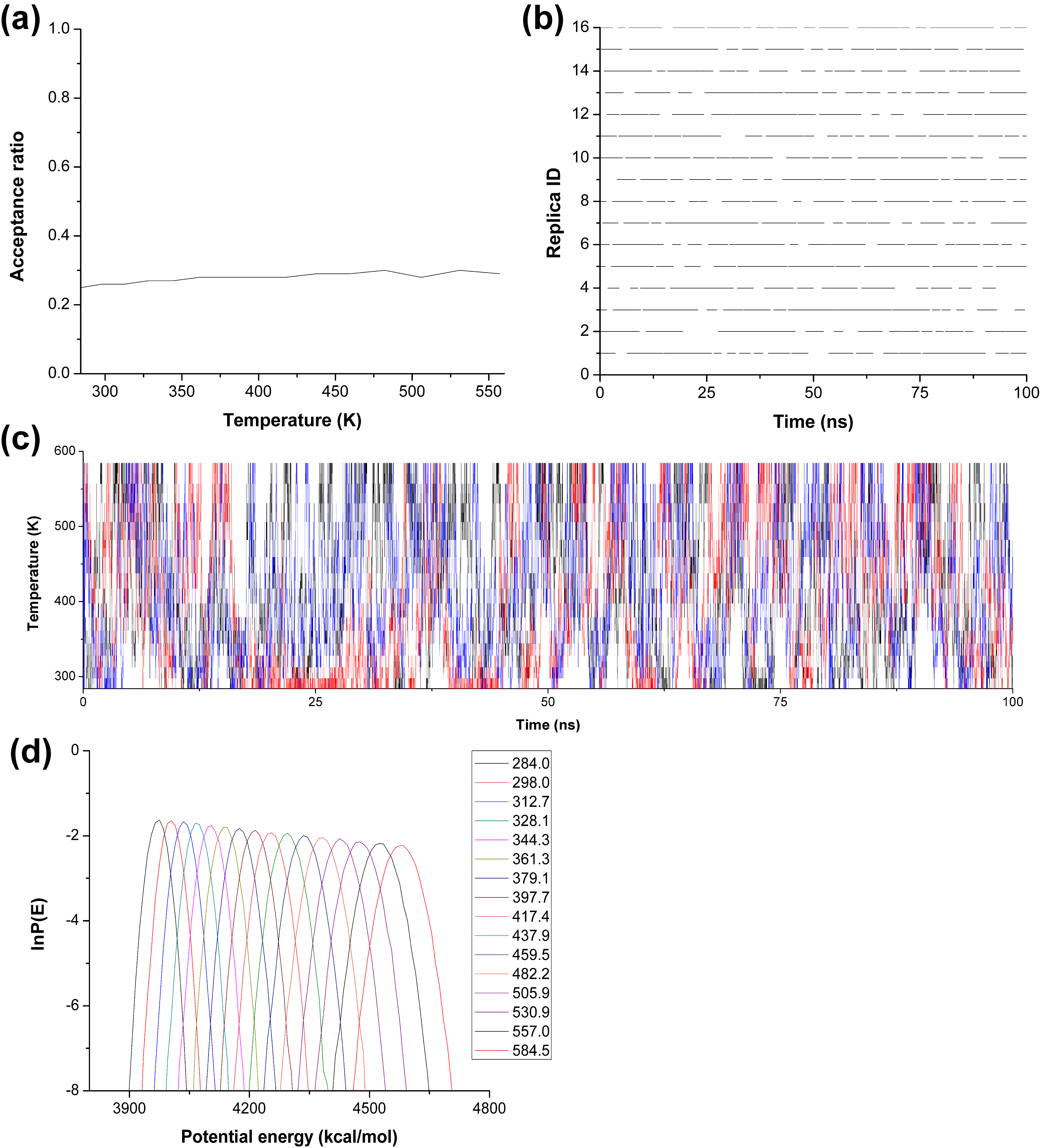

Supplement: S1 Fig — (a) Acceptance ratio of replica exchange of adjacent pairs in simulations of L34B5 in the GBHCT model. (b) Replica exchange at 298 K. (c) Time series of temperature exchange of three arbitrarily chosen replicas 1 (black), 9 (red) and 15 (blue). (d) Canonical probability of total potential energy of 16 temperatures for simulations in GBHCT model. (TIF) [file pone.0202578.s001.tif]

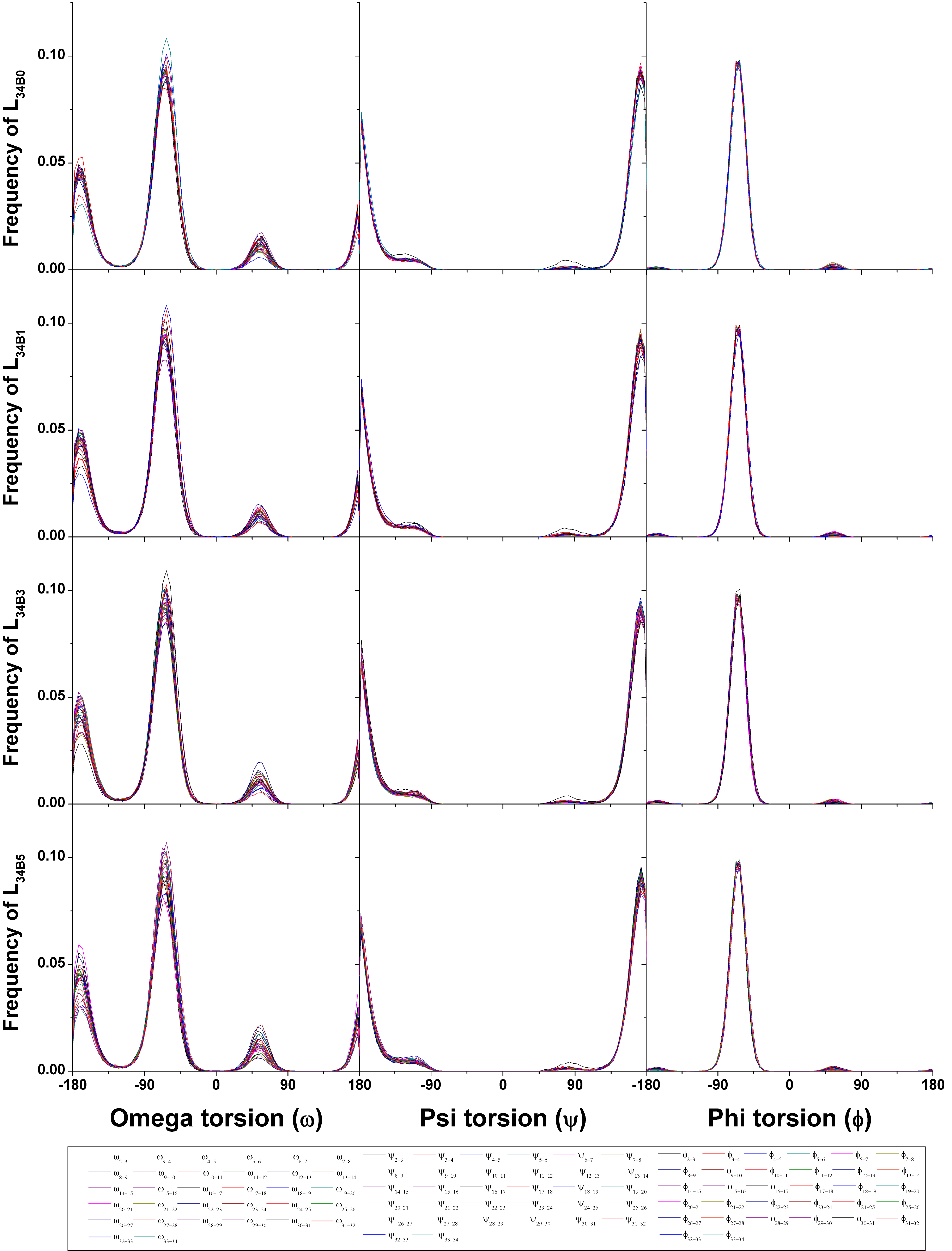

Supplement: S2 Fig — Each dihedral angle is shown in different color. (TIF) [file pone.0202578.s002.tif]
