# Supplementary material for: Unraveling the structural and molecular properties of 34-residue levans with various branching degrees by replica exchange molecular dynamics simulations
Source: PLoS One. 2018 Aug 21;13(8):e0202578. doi: 10.1371/journal.pone.0202578 (PMC6103501; doi:10.1371/journal.pone.0202578)
Supplement: S1 Table — Ranges, highest frequencies, averages of LC21 and angle3tc21 are also shown. (DOC) [file pone.0202578.s003.doc]

**S1 Table.** Populations of major representative conformers of L_34B0_, L_34B1_, L_34B3_ and L_34B5_ simulated in GB_OBC1_ model. Ranges, highest frequencies, averages of LC_21_ and angle_3tc21_ are also shown.

| Solvent model | Branch number | Number of kink | Population (%) | LC_21_ (Å) | | | Angle_3tc21_ (°) | | |
| --- | --- | --- | --- | --- | --- | --- | --- | --- | --- |
|  |  |  |  | Range | Highest frequency (frequency) | Average (s.e.m.) | Range | Highest frequency (frequency) | Average (s.e.m.) |
| GB_OBC1_ | 0  (L_34B0_) | 0 | 5.1 | 22-70 | 54 (431) | 52.4 (0.1) | 45-180 | 155 (3915) | 144.4 (0.1) |
|  |  | 1 | 26.4 | 18-66 | 50 (1599) | 46.5 (0.1) | 30-180 | 145 (15723) | 135.0 (0.1) |
|  |  | 2 | 40.9 | 16-66 | 46 (2317) | 42.5 (0.1) | 25-180 | 130 (20000) | 125.7 (0.1) |
|  |  | 3 | 23.2 | 14-62 | 44 (1371) | 39.9 (0.1) | 25-180 | 120 (13139) | 117.1 (0.1) |
|  |  | 4 | 4.1 | 14-58 | 40 (279) | 38.1 (0.1) | 30-180 | 120 (2776) | 108.6 (0.2) |
|  |  | 5 | 0.3 | 26-50 | 38 (24) | 37.0 (0.4) | 35-180 | 115 (186) | 100.3 (0.5) |
|  | 1  (L_34B1_) | 0 | 5.5 | 29-69 | 55 (216) | 52.4 (0.1) | 45-180 | 155 (4096) | 143.9 (0.1) |
|  |  | 1 | 29.0 | 18-66 | 48 (963) | 46.7 (0.1) | 30-180 | 150 (18619) | 135.2 (0.1) |
|  |  | 2 | 41.1 | 15-64 | 46 (1178) | 43.1 (0.1) | 20-180 | 130 (19944) | 126.2 (0.1) |
|  |  | 3 | 20.8 | 14-62 | 44 (665) | 40.7 (0.1) | 20-180 | 120 (12192) | 117.4 (0.1) |
|  |  | 4 | 3.5 | 16-56 | 43 (118) | 38.1 (0.2) | 25-180 | 120 (2385) | 108.2 (0.2) |
|  |  | 5 | 0.1 | 24-50 | 38 (9) | 35.0 (0.8) | 35-180 | 115 (82) | 100.0 (0.9) |
|  | 3  (L_34B3_) | 0 | 7.5 | 26-68 | 56 (535) | 52.3 (0.1) | 45-180 | 155 (5586) | 144.2 (0.1) |
|  |  | 1 | 33.3 | 20-66 | 50 (2154) | 47.1 (0.1) | 30-180 | 150 (20179) | 135.7 (0.1) |
|  |  | 2 | 40.1 | 14-64 | 46 (2300) | 43.5 (0.1) | 25-180 | 130 (19434) | 126.9 (0.1) |
|  |  | 3 | 16.8 | 14-62 | 42 (1005) | 41.0 (0.1) | 25-180 | 120 (10258) | 118.2 (0.1) |
|  |  | 4 | 2.2 | 16-58 | 40 (135) | 38.9 (0.2) | 25-180 | 120 (1538) | 109.4 (0.2) |
|  |  | 5 | 0.1 | 30-46 | 4 (8) | 37.5 (0.7) | 25-180 | 120 (51) | 98.4 (1.1) |
|  | 5  (L_34B5_) | 0 | 8.2 | 28-68 | 54 (556) | 53.1 (0.1) | 45-180 | 150 (6316) | 144.6 (0.1) |
|  |  | 1 | 34.8 | 20-70 | 50 (2053) | 47.2 (0.1) | 30-180 | 150 (21164) | 135.7 (0.1) |
|  |  | 2 | 38.4 | 20-70 | 48 (2008) | 43.8 (0.1) | 30-180 | 135 (18612) | 126.9 (0.1) |
|  |  | 3 | 16.2 | 20-66 | 44 (964) | 41.6 (0.1) | 20-180 | 120 (9917) | 118.3 (0.1) |
|  |  | 4 | 2.3 | 20-58 | 42 (153) | 39.4 (0.2) | 30-180 | 120 (1590) | 108.7 (0.2) |
|  |  | 5 | 0.1 | 26-44 | 40 (6) | 37.7 (0.9) | 50-175 | 115 (40) | 104.2 (1.2) |
