# Supplementary material for: Unraveling the structural and molecular properties of 34-residue levans with various branching degrees by replica exchange molecular dynamics simulations
Source: PLoS One. 2018 Aug 21;13(8):e0202578. doi: 10.1371/journal.pone.0202578 (PMC6103501; doi:10.1371/journal.pone.0202578)
Supplement: S2 Table — (DOC) [file pone.0202578.s004.doc]

**S2 Table.** Conformational distributions of the first and last 50 ns of the replica exchange molecular dynamics simulations of L_34B5_ in GB_HCT_ and GB_OBC1_ models.

| Solvent model | Number of branch | Number of kink | Population (%) | |
| --- | --- | --- | --- | --- |
|  |  |  | First 50 ns | Last 50 ns |
| GB_HCT_ | 5  (L_34B5_) | 0 | 7.7 | 7.4 |
|  |  | 1 | 28.5 | 27.5 |
|  |  | 2 | 35.5 | 37.9 |
|  |  | 3 | 21.4 | 22.4 |
|  |  | 4 | 6.4 | 4.6 |
|  |  | 5 | 0.5 | 0.2 |
| GB_OBC1_ | 5  (L_34B5_) | 0 | 7.7 | 8.7 |
|  |  | 1 | 35.1 | 34.4 |
|  |  | 2 | 39.0 | 37.7 |
|  |  | 3 | 15.9 | 16.6 |
|  |  | 4 | 2.2 | 2.5 |
|  |  | 5 | 0.1 | 0.1 |
