# Supplementary material for: Unraveling the structural and molecular properties of 34-residue levans with various branching degrees by replica exchange molecular dynamics simulations
Source: PLoS One. 2018 Aug 21;13(8):e0202578. doi: 10.1371/journal.pone.0202578 (PMC6103501; doi:10.1371/journal.pone.0202578)
Supplement: S3 Table — (DOC) [file pone.0202578.s005.doc]

**S3 Table.** Occurrence frequency of hydrogen bond per structure of L_34B0_, L_34B1_, L_34B3_ and L_34B5_ simulated in the GB_OBC1_ model.

| Solvent model | Branch number | Number of kinks | Occurrence frequency of hydrogen bonds per structure (%)* | | |
| --- | --- | --- | --- | --- | --- |
|  |  |  | Between residue i, i | | Between residue i, (i+1) |
|  |  |  | O5_(i)_--H1O_(i)_ | O1_(i)_--H3O_(i)_ | O6_(i)_--H3O_(i+1)_ |
| GB_OBC1_ | 0  (L_34B0_) | 0 | 6.6 | 7.5 | 31.2 |
|  |  | 1 | 6.6 | 8.0 | 30.4 |
|  |  | 2 | 6.5 | 7.8 | 29.9 |
|  |  | 3 | 6.5 | 7.7 | 29.6 |
|  |  | 4 | 6.9 | 8.1 | 29.4 |
|  |  | 5 | 6.1 | 8.2 | 30.1 |
|  | 1  (L_34B1_) | 0 | 6.5 | 8.6 | 31.1 |
|  |  | 1 | 6.3 | 8.3 | 31.1 |
|  |  | 2 | 6.4 | 8.0 | 30.8 |
|  |  | 3 | 6.3 | 8.1 | 30.4 |
|  |  | 4 | 6.4 | 8.1 | 29.9 |
|  |  | 5 | 6.0 | 8.0 | 32.0 |
|  | 3  (L_34B3_) | 0 | 5.5 | 8.9 | 32.2 |
|  |  | 1 | 5.7 | 8.7 | 32.4 |
|  |  | 2 | 5.7 | 8.7 | 32.0 |
|  |  | 3 | 5.9 | 9.0 | 31.4 |
|  |  | 4 | 6.4 | 8.6 | 31.1 |
|  |  | 5 | 5.1 | 7.9 | 32.3 |
|  | 5  (L_34B5_) | 0 | 5.0 | 9.8 | 33.4 |
|  |  | 1 | 5.1 | 9.4 | 32.9 |
|  |  | 2 | 5.2 | 9.2 | 32.5 |
|  |  | 3 | 5.3 | 9.2 | 31.9 |
|  |  | 4 | 5.3 | 9.0 | 32.1 |
|  |  | 5 | 3.4 | 11.0 | 32.9 |

*Only hydrogen bonds with the occurrence frequency per structure of at least 3% are shown.
