# Supplementary material for: Unraveling the structural and molecular properties of 34-residue levans with various branching degrees by replica exchange molecular dynamics simulations
Source: PLoS One. 2018 Aug 21;13(8):e0202578. doi: 10.1371/journal.pone.0202578 (PMC6103501; doi:10.1371/journal.pone.0202578)
Supplement: S4 Table — (DOC) [file pone.0202578.s006.doc]

**S4 Table.** Occurrence frequency of hydrogen bonds involved with branching residues of L_34B1_, L_34B3_ and L_34B5_ simulated in the GB_OBC1_ model.

| Solvent model | Branch number | Branching position | Occurrence frequency of hydrogen bonds (%)* | | | | | |
| --- | --- | --- | --- | --- | --- | --- | --- | --- |
|  |  |  | Between the same residue | | With other residues | | | |
|  |  |  | O1_(br)_--H3O_(br)_ | O5_(br)_--H1O_(br)_ | O1_(bp)_--H3O_(br)_ | O5_(bp)_--H6O_(br)_ | O3_(bp-2)_--H6O_(br)_ | O4_(bp-3)_--H1O_(br)_ |
| GB_OBC1_ | 1  (L_34B1_) | 17 | 0.29 | 0.20 | 0.59 | 0.25 | 0.13 | 0.15 |
|  | 3  (L_34B3_) | 8 | 0.27 | 0.19 | 0.61 | 0.25 | 0.13 | 0.14 |
|  |  | 16 | 0.27 | 0.19 | 0.60 | 0.26 | 0.14 | 0.16 |
|  |  | 24 | 0.29 | 0.18 | 0.60 | 0.27 | 0.14 | 0.14 |
|  | 5  (L_34B5_) | 5 | 0.28 | 0.18 | 0.59 | 0.23 | 0.15 | 0.15 |
|  |  | 10 | 0.31 | 0.17 | 0.59 | 0.30 | 0.16 | 0.16 |
|  |  | 15 | 0.29 | 0.18 | 0.59 | 0.27 | 0.16 | 0.16 |
|  |  | 20 | 0.29 | 0.18 | 0.58 | 0.27 | 0.16 | 0.15 |
|  |  | 25 | 0.28 | 0.17 | 0.60 | 0.27 | 0.13 | 0.14 |

*Only hydrogen bonds with the occurrence frequency of at least 0.05% are shown.
